# Efficient Bounded Model Checking of Heap-Manipulating Programs using Tight Field Bounds

**DOI:** 10.1007/978-3-030-71500-7_11

**Published:** 2021-02-24

**Authors:** Pablo Ponzio, Ariel Godio, Nicolás Rosner, Marcelo Arroyo, Nazareno Aguirre, Marcelo F. Frias

**Affiliations:** 8 Universidad Autónoma de Madrid, Madrid, Spain; 9 University of Twente, Enschede, The Netherlands; 10 University of Río Cuarto, Río Cuarto, Argentina; 11 Buenos Aires Institute of Technology (ITBA), Buenos Aires, Argentina; 12 National Council for Scientific and Technical Research (CONICET), Buenos Aires, Argentina

## Abstract

Software model checkers are able to exhaustively explore different bounded program executions arising from various sources of non-determinism. These tools provide statements to produce non-deterministic values for certain variables, thus forcing the corresponding model checker to consider *all* possible values for these during verification. While these statements offer an effective way of verifying programs handling basic data types and simple structured types, they are inappropriate as a mechanism for nondeterministic generation of pointers, favoring the use of insertion routines to produce dynamic data structures when verifying, via model checking, programs handling such data types.

We present a technique to improve model checking of programs handling heap-allocated data types, by taming the explosion of candidate structures that can be built when non-deterministically initializing heap object fields. The technique exploits precomputed *relational bounds*, that disregard values deemed invalid by the structure’s type invariant, thus reducing the state space to be explored by the model checker. Precomputing the relational bounds is a challenging costly task too, for which we also present an efficient algorithm, based on incremental SAT solving.

We implement our approach on top of the CBMC bounded model checker, and show that, for a number of data structures implementations, we can handle significantly larger input structures and detect faults that CBMC is unable to detect.

### 1 Introduction

SAT-based bounded model checking [7] is an automated software analysis technique, consisting of appropriately encoding a program as a propositional formula in such a way that its satisfying valuations correspond to program defects, such

* Nicolás Rosner was affiliated with the University of Buenos Aires, Buenos Aires, Argentina at the time of contribution to this work.

as violations of assertions, uncaught exceptions and memory leaks. Satisfying valuations of the obtained propositional formulas can be automatically searched for by resorting to SAT solving, exploiting the constant advances in this analysis technology. SAT-based bounded model checking achieves full automation in program verification at the cost of completeness: it limits the number of times that loops are allowed to be executed to a user provided loop unwinding bound. This in turn limits the data that the program can manipulate, which is constrained to the program parameters, and what the program can allocate in its bounded executions. Hence, although the approach is capable of exploring a huge number of execution traces, it cannot prove program correctness due to its bounded nature. Nevertheless, it is very useful for bug finding, and is able to support fully-fledged higher-level programming languages [8].

A tool based on bounded model checking over SAT is CBMC [20]. It supports all of ANSI-C, including programs handling pointers and pointer arithmetic. The tool is able to exhaustively explore many user-bounded program executions resulting from various sources of non-determinism, including scheduling decisions and the assignment of values to program variables. To achieve this, CBMC provides statements to produce non-deterministic values for certain variables, forcing the model checker to consider *all* possible values for these variables during verification. These statements enable program verification on *all* legal inputs, by assigning these inputs values within their corresponding (legal) domains. While this mechanism is effective for the verification of programs manipulating basic data types and simple structured types, it is disabled as a feature for the generation of pointers. This issue forces the user to provide an ad-hoc environment to verify programs handling dynamic data structures. In fact, a typical, convenient mechanism to verify programs handling heap-allocated linked structures using CBMC and similar tools, is to non-deterministically build such structures using insertion routines [19, 22, 11].

The aforementioned approach, while effective, has its scalability tied to how complex the insertion routines are, and how many of these are actually needed. Indeed, there are many linked structures whose domain of valid structures cannot be built only via insertion operations (e.g., red-black trees and node caching linked lists require insertions as well as removals, in order to reach all bounded valid structures). In this paper, we study an alternative technique for verifying, using CBMC, programs handling heap-allocated linked structures. The approach essentially consists of building a pool of objects with nondeterministically initialized fields, which are then used for nondeterministically building structures. The rapid explosion in the number of generated linked structures is tamed by exploiting precomputed bounds for fields, that disregard values deemed invalid by the structure's assumed properties, such as datatype invariants and routine preconditions. This leaves us the additional problem of precomputing these bounds, a computationally costly task on its own. We then present a novel algorithm for these precomputations, based on incremental SAT solving, making the whole process fully automated.

```
avl_init(t);
int size = nondet_int();
__CPROVER_assume(size>=0 && size<=MAX_SIZE);
for (int i = 0; i < size; i++) {
    int value = nondet_int();
    __CPROVER_assume(value >= MIN_VAL && value < MAX_VAL);
    avl_insert(t, value);
}
int r_value = nondet_int();
__CPROVER_assume(r_value >= MIN_VAL && r_value < MAX_VAL);
avl_remove(t, r_value);
__CPROVER_assert(avl_repok(t));
```

Fig. 1: Verification of AVL remove, building structures by multiple insertions.

We perform an experimental evaluation on a benchmark of data structure implementations, showing that the use of field bounds contributes significantly to improve both memory consumption and verification running times (including the precomputations), allowing CBMC to consider larger structures as well as to detect faults that could not be detected without their use.

### 2 A Motivating Example

Let us start by describing a particular verification scenario, that will serve the purpose of motivating our approach. Suppose that we have an implementation of dictionaries, based on AVL trees; furthermore, we would like to verify that the `remove` operation on this structure preserves the structure's invariant, i.e., after a removal is performed, the resulting structure is still a valid AVL tree (acyclic, with every node having at most one parent, sorted, and balanced). Moreover, let us assume that, besides operation `avl_remove`, we have AVL's `avl_init`, `avl_insert` and `avl_repok`, the latter being a routine that checks whether a given structure satisfies the AVL invariant, as described above. In order to perform the desired verification, we can proceed by building the program shown in Figure 1. Notice how this program:

- employs CBMC primitives to nondeterministically decide how many values, and which values to insert in/remove from the tree (appropriately constrained by constants `MAX_SIZE`, `MIN_VAL` and `MAX_VAL`),
- uses an AVL insertion routine to produce the insertions, and
- uses an `avl_repok` routine, which checks the AVL invariant on the linked structure rooted at `t`.

When running CBMC on this program, if loops are unwound enough and no violation of the assertion is obtained, then we have verified that, within the provided bounds, `remove` indeed preserves the invariant.

The above traditional approach to verifying linked structures using CBMC and similar tools [19, 22, 11] has its efficiency tied to how complex the involved routines are, in particular the insertion routine(s) (the `avl_remove` routine, being verified, cannot be avoided).

```
t = nondet_avl(MAX_SIZE, MIN_VAL, MAX_VAL);
__CPROVER_assume(avl_repok(t));
int r_value = nondet_int();
__CPROVER_assume(r_value >= MIN_VAL
  && r_value < MAX_VAL);
avl_remove(t, r_value);
__CPROVER_assert(avl_repok(t));
```

```
avlnode* nondet_avl(int size,
    int min_val,
    int max_val) {
  avlnode *n = malloc(sizeof(avlnode) * size);
  avlnode *result = NULL;
  if (nondet_bool())
    // root is null
    return result;
  result = n[0]; // root is n0
  n[0]->left = NULL;
  if (nondet_bool())
    n[0]->left = n[1];
  n[0]->right = NULL;
  if (nondet_bool())
    n[0]->right = n[1];
  else if (nondet_bool())
    n[0]->right = n[2];
  ...
  return result;
}
```

Fig. 2: Verification of AVL remove, nondeterministically building linked structs

An alternative approach, employed by some symbolic execution-based model checkers, notably [3, 23], consists of creating a pool of nodes, whose fields are nondeterministically set, and which are also nondeterministically used to build data structures. The process is illustrated in Figure 2. The key is in the use of a routine `nondet_avl()`, which encapsulates the generation of the linked structure. A fragment of this routine is shown at the right of Figure 2. Notice how this routine will generate invalid structures, e.g., cyclic ones. The `avl_repok(t)` assumption after the generation will take care of disregarding these invalid structures for verification. Notice how our manually written example generation routine is avoiding to use any node besides `n[0]` as the root, or any node but `n[1]` as `n[0]->left`, thus avoiding some isomorphic structures and obvious cycles, but it does not avoid nodes from having more than one parent, nor it seems to take into account the tree's balancedness. Of course, we have other alternatives when writing the nondeterministic generation routine `nondet_avl`. We may produce a generation routine that, based solely on the fields of the nodes involved in the structure and their types, produces all possible structures, leaving the work of filtering out valid ones to the `assume(avl_repok(t))` part of the program. We can also write a sophisticated generation routine specifically tailored for AVL trees, that already takes into account (most) invalid values for each node field, and thus mostly produces valid structures. The first option has as an advantage that it is *generic*, and thus can be made part of an automated verification technique, at the cost of being, intuitively, less efficient; the second (and our example), on the other hand, has in principle to be manually produced, and is more error prone, since we may be disregarding some valid values making the verification bounded incomplete, but is intuitively more efficient.

The technique we present in this paper consists of automatically producing the second kind of generation routines. We will start with the first kind of generation, and automatically decide which values for each field of each node can be safely removed, when we can establish that they do not participate in valid structures (i.e., structures satisfying the corresponding structure invariant). This additional problem of deciding when a value for a node field's domain can be safely removed is solved using a novel algorithm, presented in this paper, which uses incremental SAT solving.

### 3 Tight Field Bounds

Tight field bounds are based on a relational semantics of structures' fields in program states. The relational semantics of structures is based on interpreting a field `f` at a given program state as the set of pairs $\langle id, v\rangle$ relating the identifier $id$ (representing a unique reference to some data object $o$ in the heap) with the value $v$ in the field $f$ of $o$ at that state (i.e., $o$`->f` $= v$ in the state). Then, each program state corresponds to a set of (functional) binary relations, one per field of the structures involved in the program. For example, the program state containing the binary tree depicted at the left of Fig. 3 are represented by the following relations:

$$\texttt{left} = \{\langle N0, N1\rangle\ \langle N1, N3\rangle\},\ \ \texttt{right} = \{\langle N0, N2\rangle\ \langle N1, N4\rangle, \langle N2, N5\rangle\} \qquad (1)$$

For analysis techniques that must consider all possible state configurations that satisfy some given property, we may reduce this relational semantics by considering *tight field bounds*. Intuitively, for a field $f$ and a property $\alpha$, its tight field bound on $\alpha$ is the union of $f$'s representation across all program states that satisfy $\alpha$. Tight field bounds have been used to reduce the number of variables and clauses in propositional representations of relational heap encodings for Java automated analyses [14, 13, 2], and in symbolic execution based model checking to prune parts of the symbolic execution search tree constraining nondeterministic options [15, 26] (see section 6 for a more detailed description of these previous applications). Tight field bounds are computed from *assumed* properties, and can be employed to restrict structures in states that are assumed to satisfy such properties, i.e. *precondition* states. In our case, we will use the invariant of the structure, as opposed to stronger preconditions, so that these can be reused across several routines of the same structure.

**Definition 1.** *Let $f$ be a field of structure $T_1$ with type $T_2$. Let $i$ and $j$ be the scopes for types $T_1$ and $T_2$, respectively. Let $A = \{a_1, \ldots, a_i\}$ be the identifiers for data objects of type $T_1$, and let $B = \{b_1, \ldots, b_j\}$ be the identifiers for data objects of type $T_2$. Given an identifier $k$, $o_k$ denotes the corresponding data object. The tight field bound for field $f$ is the smallest relation $U_f \subseteq A \times (B + Null)$ satisfying: $\langle x, y\rangle \in U_f$ iff there exists a valid heap instance $I$ in which $o_x$->$f = o_y$.*

By *scope* we mean the limit in the number of objects, ranges for numerical types, and maximum depth in loop unwinding, as in [17, 12]. An important assumption we make for analysis is that structure invariants do not refer to the specific heap addresses of data objects, and in particular that these do not

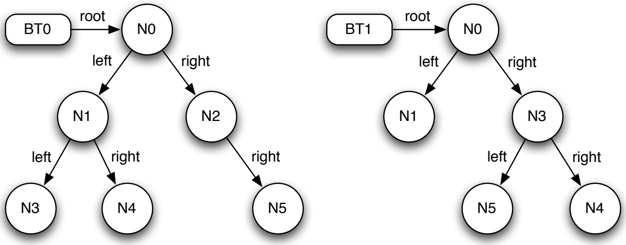

Fig. 3: Two valid binary trees.

use pointer arithmetic. Therefore, permuting data object identifiers on a valid instance still yields a valid instance (i.e., permuting the actual locations of data objects in the heap is irrelevant for invariant satisfaction). This is most times the case, and is indeed the case in all the examples that we will present in Section 5. This is an important assumption because it enables us to add an additional implicit invariant: *symmetry breaking*. This has an important impact in the size of tight field bounds, since they get greatly reduced when isomorphic structures are removed. We use a symmetry breaking procedure that removes all symmetries. For details, we refer the reader to [14, 13].

## 4 A Technique for Nondeterministic Generation of Dynamic Structures

We are now ready to describe the technique for nondeterministic generation of dynamic structures, used to verify programs handling such data using CBMC. The technique requires:

- the program `p(T x)` to be analyzed;
- a description of the structure of type `T`, which in the dynamic case, typically consists of a struct or set of structs (that are dynamically allocated);
- a boolean program `repok(T x)`, that (operationally) decides whether a given instance `x` is valid, i.e., satisfies the structure's invariant, or not; and
- a tight field bound $B_f$ for every field $f$ in `T` and the scope $n$ to use for bounded model checking of $p$.

The first three are necessary information; for the last one we present later on in the paper an algorithm to compute tight bounds, from the other three.

The technique starts by building a routine `nondet_T()`, that produces and returns structures of type `T`. The routine works as follows. First, for every (pointer) type `Tt` involved (including `T`), we start by allocating $n$ (the scope) data objects:

```
Tt *tt_nodes = malloc(sizeof(Tt) * n);
```

Then, for every structure pointer type `Ts` (for which we already allocated $n$ data objects) and field `f` of type `Tt` in `Ts`, we build the following nondeterministic assignment:

```
ts_nodes[0]->f = NULL;
if (nondet_bool()) ts_nodes[0]->f = tt_nodes[0];
else if (nondet_bool()) ts_nodes[0]->f = tt_nodes[1];
...
ts_nodes[1]->f = NULL;
if (nondet_bool()) ts_nodes[1]->f = tt_nodes[0];
else if (nondet_bool()) ts_nodes[1]->f = tt_nodes[1];
...
```

Finally, `nondet_T()` ends by returning either `NULL` or `t_nodes[0]` (no other non-null node is necessary, due to symmetry breaking). Using `nondet_T()`, we build the following verification harness for `p`:

```
T x = nondet_T();
__CPROVER_assume(repok(x));
p(x);
__CPROVER_assert(repok(x));
```

Of course the last assertion can be replaced by any expected property of `p`.

We now turn our attention to the use of tight field bounds to reduce non-determinism in `nondet_T()`. For every structure `Ts` and field `f` with type `Tt` declared in `Ts`, if $\langle N_i^{Ts} N_j^{Tt} \rangle$ does not belong to the tight bound $B_f$, then we remove from `nondet_T()` the line:

```
if (nondet_bool()) ts_nodes[i]->f = tt_nodes[j];
```

To illustrate the benefits of using tight field bounds in this setting, compare the two (semantically equivalent) `nondet_avl()` methods in Figure 4 for building AVLs with size at most 4. At the left of Figure 4, we show the code for the approach that considers all the feasible assignments to nodes' fields within the scope (many assignments not displayed due to the lack of space). With precomputed tight field bounds we can discard a significant number of these assignments, that are not allowed due to the bounds, as shown at the right of Figure 4. Notice that, among many others, all self-loops in nodes are discarded by the bounds.

#### 4.1 Computing Tight Field Bounds

For the rest of this section we assume a fixed structure `T`, with fields $f_1, \ldots, f_m$ and representation invariant `repok`, and a fixed scope `k`. Tight field bounds for `T` can be automatically computed from assumed properties such as invariants and preconditions. These properties must be expressed in a language amenable to automated analysis, reducible to SAT-based analysis in our case. We employ the automated translation of the definition of `T` and its `repok` to a propositional formula implemented in the TACO tool [14, 13]. We also assume a symmetry breaking predicate is created by this translation, forcing canonical orderings of heap nodes in structures (see [14, 13] for a careful description of how these symmetry-breaking predicates are automatically built). We discuss below the

```
avlnode* nondet_avl() {
  avlnode *n = malloc(sizeof(avlnode)*4);
  if (nondet_bool())
    return NULL;
  avlnode *result = n[0];
  // assignments to n[0]'s fields
  n[0]->left = NULL;
  if (nondet_bool())
    n[0]->left = n[0];
  else if (nondet_bool())
    n[0]->left = n[1];
  else if (nondet_bool())
    n[0]->left = n[2];
  else if (nondet_bool())
    n[0]->left = n[3];
  n[0]->right = NULL;
  if (nondet_bool())
    n[0]->right = n[0];
  else if (nondet_bool())
    n[0]->right = n[1];
  else if (nondet_bool())
    n[0]->right = n[2];
  else if (nondet_bool())
    n[0]->right = n[3];
  n[0]->height = 0;
  if (nondet_bool())
    n[0]->height = 1;
  else if (nondet_bool())
    n[0]->height = 2;
  else if (nondet_bool())
    n[0]->height = 3;
  // assignments to n[1], n[2] and n[3]'s
  // fields follow a similar pattern to
  // n[0]'s and are ommited
  return result;
}
```

```
avlnode* nondet_avl() {
  avlnode *n = malloc(sizeof(avlnode)*4);
  if (nondet_bool()) return NULL;
  avlnode *result = n[0];
  // assignments to n[0]'s fields
  n[0]->left = NULL;
  if (nondet_bool())
    n[0]->left = n[1];
  n[0]->right = NULL;
  if (nondet_bool())
    n[0]->right = n[1];
  else if (nondet_bool())
    n[0]->right = n[2];
  n[0]->height = 1;
  if (nondet_bool())
    n[0]->height = 2;
  else if (nondet_bool())
    n[0]->height = 3;
  // assignments to n[1]'s fields
  n[1]->left = NULL;
  if (nondet_bool())
    n[1]->left = n[3];
  n[1]->right = NULL;
  if (nondet_bool())
    n[1]->right = n[3];
  n[1]->height = 1;
  if (nondet_bool())
    n[1]->height = 2;
  // assignments to n[2]'s fields
  n[2]->left = NULL;
  if (nondet_bool())
    n[2]->left = n[3];
  n[2]->right = NULL;
  if (nondet_bool())
    n[2]->right = n[3];
  n[2]->height = 1;
  if (nondet_bool())
    n[2]->height = 2;
  // assignments to n[3]'s fields
  n[3]->left = NULL;
  n[3]->right = NULL;
  n[3]->height = 1;
  return result; }
```

Fig. 4: Building AVLs with size at most 4. Left: all feasible assignments to node's fields. Right: only assignments deemed feasible by tight field bounds parts of the translation that are important for the understanding of our approach, and refer the reader to the literature for additional details [14, 13].

Let `f` be a field of `T` with type `T'`. Let $A = a_1, \ldots, a_k$ and $B = b_1, \ldots, b_k$ be the identifiers for data objects of type `T` and `T'` within scope `k`, respectively. This bounded field is then a relation $f \subseteq A \times (B + null)$. The propositional encoding of $f$ consists of boolean variables $f_{i,j}$, $0 \leq i, j < k$, such that $f_{i,j} = True$ in a instance $I$ if and only if the value of $f$ for object $a_i$ is equal to object $b_j$ (i.e. $a_i$`->`$f = b_j$) in $I$ (the original translation has variables representing $a_i$`->`$f = null$, we omit these here to simplify the presentation).

As an example, Figure 5 below shows the propositional variables representing all the feasible values of binary trees' `left` and `right` fields for scope 6, in tabular form. In the tables, object identifiers are named $N_i$ $(0 \leq i < 6)$, variables $l_{i,j}$ $(0 \leq i, j < 6)$ denote $N_i$->$left = N_j$ (similarly, $r_{i,j}$ denote $N_i$->$right = N_j$).

| left | $N_0$ | $N_1$ | $\dots$ | $N_5$ |
|---|---|---|---|---|
| $N_0$ | $l_{0,0}$ | $l_{0,1}$ | $\dots$ | $l_{0,5}$ |
| $N_1$ | $l_{1,0}$ | $l_{1,1}$ | $\dots$ | $l_{1,5}$ |
| $\vdots$ | $\vdots$ | $\vdots$ | $\ddots$ | $\vdots$ |
| $N_5$ | $l_{5,0}$ | $l_{5,1}$ | $\dots$ | $l_{5,5}$ |

| right | $N_0$ | $N_1$ | $\dots$ | $N_5$ |
|---|---|---|---|---|
| $N_0$ | $r_{0,0}$ | $r_{0,1}$ | $\dots$ | $r_{0,5}$ |
| $N_1$ | $r_{1,0}$ | $r_{1,1}$ | $\dots$ | $r_{1,5}$ |
| $\vdots$ | $\vdots$ | $\vdots$ | $\ddots$ | $\vdots$ |
| $N_5$ | $r_{5,0}$ | $r_{5,1}$ | $\dots$ | $r_{5,5}$ |

Fig. 5: Propositional encodings of binary trees' `left` and `right` fields for a scope of 6

In this way, the binary tree at the left of Figure 3, whose relational representation is given in equation 1, is defined exactly by setting the following variables to true (and all the remaining variables to false):

$$\texttt{left} = \{l_{0,1}, l_{1,3}\}, \quad \texttt{right} = \{r_{0,2}, r_{1,4}, r_{2,5}\} \tag{2}$$

As each propositional variable in the encoding of a field represents exactly the fact that a single pair of objects belongs to the field, in the following we will speak of these two notions (propositional variables and pairs of objects related by a field) interchangeably. In fact, as our approach operates with propositional formulas (needed for exploiting incremental SAT solving), the tight field bounds will be represented and computed in terms of propositional variables. It is straightforward to see that if variable $f_{i,j}$ belongs to the tight field bound for field $f$, then $\langle a_i, b_j \rangle$ is a feasible pair in the relational semantics (and is infeasible if $f_{i,j}$ does not belong to the tight field bound).

It is worth noticing that deciding if there exists a structure with a particular field value, say $a_i$->$f = b_j$, can be accomplished by querying the solver about the satisfiability of a formula consisting of a propositional encoding of the structure and the invariant (`prop_repok`), the propositional encoding of the symmetry breaking predicate (`prop_sbpred`), and the corresponding variable $f_{i,j}$:

$$\texttt{prop\_repok} \wedge \texttt{prop\_sbpred} \wedge f_{i,j} \tag{3}$$

In case the satisfiability verdict is true, the valuation returned by the solver corresponds to a *valid* (in the sense that it satisfies the invariant) memory heap, containing pair $\langle a_i, b_j \rangle$ in the relational representation of $f$. Also, from the valuation we can retrieve for each field $f$ all the (true) variables that represent pairs of objects related by $f$ in that particular heap.

The formula above can be used to compute tight bounds, determining what are the infeasible variables $f_{i,j}$ (and hence the corresponding pairs in the fields' semantics), in states that satisfy the invariant. In [14], the infeasible variables are determined using a top-down algorithm. In the algorithm therein, the field semantics is initially set, for a field of type $B$ declared in structure $A$, to $A \times$

$(B \cup \{null\})$. From this fully populated initial semantics, each pair is checked for feasibility. Pairs found to be infeasible are removed from the bound. Adopting this top-down approach for computing tight field bounds leads to feasibility checks (a large number of these) that are *independent* from one another, thus making it amenable to distributed processing. Moreover, a pair can be removed from the bound as soon as it is deemed infeasible, which can be exploited to compute tight field bounds "non-exhaustively", e.g., dedicating a certain time to the computation of tight field bounds, and taking the obtained tight field bound for improving SAT analysis, regardless of whether the tight bound is the *tightest* (it converged to removing all infeasible pairs) or not. The latter can be achieved thanks to the fact that, in the top-down approach, intermediate bounds are also tight bounds [14, 13]. As each SAT query in this top-down approach is independent from the rest, the algorithm does not exploit the incremental capabilities of modern SAT solvers.

Let us present our approach to compute tight field bounds. As opposed to the technique in [14], our algorithm operates in a *bottom-up* fashion. In our presentation below, we assume a `propEncoding` method that takes the `repok`, a symmetry breaking predicate `sbpred`, and the scopes `scope`, and returns an `encoding` object. Its `getPropositionalFormula` method creates and returns a CNF propositional formula, encoding the `repok` and `sbpred` for the given `scope`. Also, the `encoding`'s `getVars(f)` method returns all the propositional variables in the encoding of field $f$ (see Figure 5). The algorithm uses an *incremental* SAT solver, represented by a module `solver`, with the following routines:

- `load`: receives as argument a propositional formula in CNF and loads it into the solver.
- `addClause`: (incrementally) adds a clause to the current formula in the solver for future solving invocations.
- `solve`: calls the SAT-solving procedure, deciding whether the formula currently loaded in the solver is satisfiable (SAT) or not.
- `getModel`: if the formula is satisfiable, it returns the valuation produced by the SAT-solver. The truth value of a variable `v` in the model can be retrieved by invoking `getValue(v)`.

The pseudocode of our algorithm is shown in Figure 6. Line 3 builds a propositional `encoding` using the `repok`, the symmetry breaking predicate `sbpred` and the `scopes`. The CNF propositional formula produced by the `encoding` object is then loaded into the `solver` in Line 4. Lines 5-7 initialize sets $\texttt{vars\_f}_1, \ldots, \texttt{vars\_f}_m$, each containing all the propositional variables in the encoding of the corresponding fields $\texttt{f}_1, \cdots, \texttt{f}_m$. As opposed to the top-down algorithm proposed in [14], which initialized fields' semantics as binary relations containing all pairs, the bottom-up algorithm starts with empty sets $\texttt{feasible\_f}_1, \ldots, \texttt{feasible\_f}_m$ (lines 8-10). $\texttt{feasible\_f}_1, \ldots, \texttt{feasible\_f}_m$ are used by the algorithm to store partial bounds for the corresponding fields $\texttt{f}_1, \cdots, \texttt{f}_m$, and will be iteratively extended with the true variables in instances returned by the SAT solver.

A crucial step in our algorithm is performed at line 12, where the current formula loaded in the SAT solver is extended, exploiting incremental SAT solving [16], with a progress-ensuring constraint on heap fields. Here, we add a clause that consists of the disjunction of all the variables in the encoding of fields that have not been previously added to the $\texttt{feasible\_f}_1, \ldots, \texttt{feasible\_f}_m$ sets. The purpose of is to ensure that instances returned by `solver.solve()` in Line 13 contain at least one pair that does not belong to the sets already held in $\texttt{feasible\_f}_1, \ldots, \texttt{feasible\_f}_m$. Intuitively, by adding the clause in line 12, the call to `solver.solve()` in line 13 can be interpreted as *"find a valid heap instance of the data structure that can be used to extend at least one of the current bounds in* $\texttt{feasible\_f}_1, \ldots, \texttt{feasible\_f}_m$*"*. If such an instance exists, it is returned by the `solver.getModel()` method, and stored in the `model` variable in line 14. The variables that are true in `model` are then added to the $\texttt{feasible\_f}_1, \ldots, \texttt{feasible\_f}_m$ sets in lines 15-19. The loop terminates when $\texttt{feasible\_f}_1, \ldots, \texttt{feasible\_f}_m$ cannot be augmented any further (lines 20, 21), in which case, as we prove below, these sets hold tight field bounds and are returned by the algorithm (line 24).

As an example, assume we are computing tight field bounds for binary trees, and that the invocation to `solver.solve()` returned the instance at the left of Figure 3. Then, the variables in sets *left* and *right* shown in equation 2 will be added to `feasible_left` and `feasible_right`, respectively, in lines 15-19. Notice that this forces an instance with at least one variable not in the `left` or `right` sets to be returned by `solver.solve()` in the next iteration.

It is worth noticing the importance of the progress-ensuring constraint in line 12, being encoded as a clause. This is what enables the possibility of using *incremental SAT solving* [16] in our tight bounds computation. Essentially, incremental SAT solvers allow one to append further constraints after each satisfying valuation is found, as long as these are in CNF. These constraints are conjoined with the main (CNF) formula, and used in computing the "next" satisfying instance without having to restart the solving process (which is a very time consuming process). Also, this allows the solver to exploit the learned clauses (that summarize the conflicts found by the solver in the search of satisfying valuations) to help accelerate subsequent queries [10]. Notice that, if the new constraints were not in CNF, the whole resulting formula would have to be translated to CNF and the SAT process restarted from scratch.

Theorem 1 proves our algorithm terminates and computes tight field bounds.

**Theorem 1.** *Algorithm 6 terminates and returns valid tight field bounds.*

*Proof.* Termination easily follows from the following two facts: *(i)* for given bounds on data domains of the structure under analysis and limited by *scopes*, the number of pairs that can be added to a field bound is a finite number; and *(ii)* each while-loop iteration either adds at least an extra pair to the bounds, or otherwise returns *unsat*, in which case the loop terminates.

To prove that the algorithm yields tight field bounds, we proceed as follows. Notice that at each iteration, and for any field $f_i$, the bound associated to field $f_i$ ($\texttt{feasible\_f}_i$) is a subset of the corresponding tight bound, i.e., contains only feasible variables: the initial bound ($\emptyset$) is obviously a subset of the tight

```
procedure bottom-up(repok, sbpred, scopes)
begin
  encoding = propEncoding(repok, sbpred, scopes)
  solver.load(encoding.getPropositionalFormula())
  vars_f1 = enconding.getVars(f1)
  ...
  vars_fm = enconding.getVars(fm)
  feasible_f1 = {}
  ...
  feasible_fm = {}
  while True do
    solver.addClause(⋁_{j∈1,..,m, v∈(vars_fj \ feasible_fj)} v)
    if solver.solve() = SAT then
      model = solver.getModel()
      feasible_f1 = feasible_f1 ∪
          {v | v <- vars_f1 and model.getValue(v) = True}
      ...
      feasible_fm = feasible_fn ∪
          {v | v <- vars_fm and model.getValue(v) = True}
    else \\ UNSAT
      break
    fi
  done
  return feasible_f1, ..., feasible_fm
end
```

Fig. 6: Bottom-up algorithm for tight field bounds computation bound, and bounds are extended only by adding variables extracted from valid structures (i.e., each loop iteration produces a valid expansion). An inductive argument allows us to conclude that, on termination, the bound associated to field $f_i$ (feasible_f$_i$) is a subset of the tight bound. We will now show that feasible_f$_i$ is the tight field bound. Let us suppose that, once the algorithm terminates, bound feasible_f$_i$ is not tight, i.e., there exists a variable $v_{w,z}$ that does not belong to feasible_f$_i$. Then, there must exist a canonical (i.e., satisfying symmetry breaking) instance $I$ of repok within scopes, in which $o_w$->$f_i = o_z$. Therefore, $I$ satisfies repok, sbpred, and $v_{w,z} = True$, contradicting the fact that the algorithm had terminated. Therefore, all variables excluded from feasible_f$_i$ are infeasible, making this bound tight.

As opposed to the top-down algorithm for tight bounds introduced in [14, 13] Algorithm 6 only provides useful information once it terminates – intermediate bounds cannot be used to improve analysis. Moreover, whereas the top-down approach lends itself well to parallelization (as we mentioned before, it implies a large number of independent SAT queries, that can be solved in a distributed manner), it is not obvious how one would reasonably distribute our new bottomup computation. Nevertheless, as we will show in Section 5, the sequential Algorithm 6 and its optimizations (i.e. the usage of incremental SAT-solving) are substantially faster than the parallel, distributed, top-down approach.

## 5 Evaluation

Our first experimental evaluation assesses the impact of tight field bounds in verification of code handling linked structures using CBMC. The evaluation is based on a benchmark of collection implementations, previously used for tight field bounds computation in [14, 13], composed of data structures with increasingly complex invariants:

- an implementation of sequences based on singly linked lists (LList);
- a List implementation (from Apache Commons.Collections), based on circular doubly-linked lists (AList);
- a List implementation (from Apache Commons Collections), based on node caching linked lists (CList);
- a Set implementation (from java.util) based on red-black trees (TSet);
- an implementation of AVL trees obtained from the case study used in [4] (AVL); and
- an implementation of binomial heaps used as part of a benchmark in [28] (BHeap).

Experiments in this section were run on workstations with Intel Core i7 4790 processor, 8Mb Cache, 3.6Ghz (4 Turbo), and 16 Gb of RAM, running GNU/Linux. The incremental SAT solver used was Minisat 2.2.0. We denote by OOM that the 16GB of memory were exhausted, and by OOM+ that the 16GB where exhausted while CBMC was preprocessing; in this latter case no numbers of clauses or variables were produced by CBMC. Timeout was set for these experiments to 1 hour.

Table 1 reports, for the most relevant routines of each of the data structures in our benchmark, the verification running times with the underlying decision procedure running times discriminated in seconds, as well as the number of clauses and variables (expressed in thousands) in the CNF formulas corresponding to each of the verification tasks, for several scopes (S). Since we checked whether the routines preserved the corresponding structure's invariant, we did not consider for the experiments those routines that did not modify the structure (these trivially preserve the invariant). We assessed three different approaches:

- Build*: use of verification harnesses based on insertion routines (see Fig. 1),
- Gen&Filter (generate and filter): non-deterministic generation of data structures without tight field bounds (as illustrated in Fig. 4), using a traditional symmetry breaking algorithm to discard isomorphic structures [14] (we do not discuss this here due to space reasons),
- TFB: our introduced approach, which incorporates tight field bounds into the previous to discard irrelevant non-deterministic assignments of field values (as illustrated in Fig. 4).

Some remarks on the results are in order. Table 1 shows that in all analyzed routines, the TFB approach allowed us to analyze larger scopes for which the other input generation techniques exhausted the allotted time or memory. TFB was able to analyze larger scopes than Gen&Filter in 7 out of 12 cases (remarkably, by at least 6 in AList, at least 3 in CList and at least 2 in AVL), and in 8 out of 12 cases with respect to Build* (by at least 4 in all 8 cases). Routine extractMin in structure BHeap is particularly interesting: it contains a bug first found in [14] that can only be exhibited by an input with at least 13 nodes. Gray cells mark experiments in which the bug was detected by CBMC. Notice in particular that Build* does not scale well enough to find this bug.

Our second evaluation is devoted to tight field bounds computation, in comparison with the top-down approach presented in [14]. We re-ran the TACO experiments as reported in [13] on the same hardware we used for our own experiments for a fair comparison. Original scripts and configurations were preserved. All distributed experiments were run on a cluster of 9 PCs (one being the master) of the same characteristics as described above. Each distributed experiment was run 3 times; the reported timing is the average thereof. All times are given in wall-clock seconds. A timeout (TO) is set at 18,000 seconds (5 hours), for tight bounds computation. Our bottom up tight field bounds technique is non-parallel, and was run on a single workstation. Table 2 summarizes the results of our experiments regarding tight bounds computation. We compared the running times of computing tight field bounds using the distributed technique from [14] and our non-parallel presented algorithm, for scopes 10, 12, 15, 17 and 20, reporting the following:

- TACO(||): The parallel wall-clock time required to compute tight field bounds with TACO, the tool subsuming the top-down tight bounds approach [14, 13].
- TACO(s): The TACO *sequentialized* time, i.e., the sum of times over all the Minisat solvings performed by the TACO distributed algorithm.
- BU: The time the bottom-up algorithm (Alg. 6) requires to compute tight field bounds.
- speedup(||): The speed-up achieved by BU when compared to the distributed TACO time reported as TACO(||).
- Speedup(s): The speed-up achieved by BU when compared to the sequentialized TACO time reported as TACO(s).

The speed-ups obtained by Alg. 6 are, in comparison with the distributed approach in [14], in general very good. In particular, in all experiments but AVL with scope 20, the running time of our sequential bottom-up approach (BU) is already below the wall-clock time of (parallel) TACO. For AVL trees with scope 20, the only experiment where BU performed slower than TACO, the achieved speed up is 0.6X. This means that running BU on a single workstation does not even take twice as long as running TACO(||) on 32 processors (4 cores in 8 slave machines used for distributed computation). Second, it is worth noticing that structures with strong invariants (e.g., BHeap) intuitively lead to "small" tight field bounds; a bottom-up approach then, as we explained earlier, is particularly well suited for tight bounds computation for these structures, since the process

Table 1: Dynamic data structure verification in CBMC: TFB versus Build* and Gen&Filter. Verification and solving times in seconds, clauses and variables in thousands

| | Routine | S | Build* | | | Gen&Filter | | | TFB | | |
|---|---|---|---|---|---|---|---|---|---|---|---|
| | | | Time(Solv) | Clauses | Vars | Time(Solv) | Clauses | Vars | Time(Solv) | Clauses | Vars |
| SList | insBack | 18 | 10(5) | 705 | 2,236 | 11(5) | 248 | 1,157 | 10(4) | 188 | 916 |
| | | 19 | 12(6) | 797 | 2,524 | 13(6) | 275 | 1,288 | 11(4) | 206 | 1,015 |
| | | 20 | 14(7) | 898 | 2,836 | 16(7) | 303 | 1,428 | 13(5) | 226 | 1,122 |
| | remove | 18 | 10(6) | 629 | 2,004 | 14(9) | 247 | 1,154 | 11(6) | 201 | 967 |
| | | 19 | 13(8) | 715 | 2,274 | 23(16) | 275 | 1,288 | 13(7) | 221 | 1,075 |
| | | 20 | 14(9) | 809 | 2,567 | 20(12) | 303 | 1,431 | 15(8) | 243 | 1,190 |
| AList | addLast | 13 | 2(1) | 146 | 628 | 9(7) | 235 | 947 | 3(2) | 184 | 738 |
| | | 14 | 2(1) | 164 | 704 | TO | 267 | 1,082 | 3(2) | 206 | 827 |
| | | 20 | 6(4) | 292 | 1,285 | – | – | – | 8(6) | 357 | 1,459 |
| | remInd | 14 | 5(3) | 255 | 1,099 | 1168(1166) | 352 | 1,444 | 8(6) | 307 | 1,270 |
| | | 15 | 6(5) | 287 | 1,238 | TO | 400 | 1,645 | 10(8) | 346 | 1,431 |
| | | 20 | 17(14) | 471 | 2,058 | – | – | – | 27/24 | 568 | 2,387 |
| CList | addLast | 6 | 407(402) | 2,471 | 9,937 | 2(1) | 109 | 430 | 1(1) | 103 | 402 |
| | | 7 | TO | 3,754 | 15,158 | 2(1) | 133 | 527 | 2(1) | 122 | 482 |
| | | 17 | – | – | – | 1423(1419) | 527 | 2,188 | 10(7) | 411 | 1,692 |
| | | 18 | – | – | – | TO | 583 | 2,425 | 10(7) | 449 | 1,853 |
| | | 20 | – | – | – | – | – | – | 14(10) | 530 | 2,195 |
| | remove | 6 | 490(486) | 1,750 | 6,994 | 4(3) | 258 | 1,002 | 4(3) | 247 | 958 |
| | | 7 | TO | 2,755 | 11,066 | 8(3) | 356 | 1,395 | 5(4) | 332 | 1,298 |
| | | 15 | – | – | – | 2820(2812) | 2,151 | 8,642 | 60(52) | 1,768 | 7,103 |
| | | 16 | – | – | – | TO | 2,537 | 10,202 | 102(93) | 2,067 | 8,315 |
| | | 20 | – | – | – | – | – | – | 219(201) | 3,578 | 14,454 |
| AVL | insert | 1 | 114(105) | 13,724 | 58,613 | 19(17) | 2,232 | 9,593 | 7(5) | 712 | 3,006 |
| | | 2 | OoM+ | – | – | 69(62) | 7,011 | 30,138 | 21(15) | 1,665 | 7,125 |
| | | 3 | – | – | – | 203(182) | 19,230 | 82,602 | 57(38) | 3,414 | 14,745 |
| | | 4 | – | – | – | OoM+ | – | – | 169(114) | 7,005 | 30,500 |
| | | 5 | – | – | – | – | – | – | 411(266) | 13,818 | 60,663 |
| | | 6 | – | – | – | – | – | – | OoM | 26,981 | 119,475 |
| | delete | 2 | 94(87) | 10,874 | 46,271 | 11(10) | 1,227 | 5,257 | 4(3) | 421 | 1,777 |
| | | 3 | OoM+ | | | 39(34) | 3,844 | 16,491 | 11(8) | 823 | 3,487 |
| | | 4 | – | – | – | 118(105) | 10,522 | 45,120 | 40(29) | 2,351 | 10,058 |
| | | 5 | – | – | – | OoM | 26,768 | 114,697 | 108(81) | 3,823 | 16,387 |
| | | 7 | – | – | – | – | – | – | 1171/1011 | 14,365 | 62,154 |
| | | 8 | – | – | – | – | – | – | OoM+ | – | – |
| TSet | add | 1 | 158(104) | 11,849 | 43,627 | 20(16) | 2,093 | 8,076 | 10(7) | 980 | 3,811 |
| | | 2 | OoM+ | – | – | 63(62) | 5,609 | 21,908 | 37(27) | 3,043 | 11,961 |
| | | 4 | – | – | – | 362(314) | 23,538 | 93,122 | 206(160) | 12,042 | 47,960 |
| | | 5 | – | – | – | OoM+ | – | – | 386(305) | 18,270 | 73,206 |
| | | 6 | – | – | – | – | – | – | OoM+ | | |
| | remove | 2 | 128(85) | 9,708 | 34,998 | 10(7) | 1,029 | 3,934 | 9(7) | 1,000 | 3,825 |
| | | 3 | OoM | 28,268 | 107,255 | 23(19) | 2,074 | 8,039 | 22(17) | 2,016 | 7,818 |
| | | 9 | – | – | – | 828/761 | 22,881 | 91,143 | 760/699 | 22,698 | 90,434 |
| | | 10 | – | – | – | OoM | 29,724 | 118,620 | OoM | 29,548 | 117,943 |
| BHeap | insert | 5 | 188(181) | 17,620 | 75,858 | 35(32) | 2,722 | 11,679 | 32(28) | 2,627 | 11,297 |
| | | 6 | OoM | 27,217 | 117,396 | 56(50) | 3,852 | 16,522 | 47(42) | 3,717 | 15,972 |
| | | 13 | – | – | – | 640/603 | 18,480 | 78,795 | 523/487 | 17,827 | 76,142 |
| | | 14 | – | – | – | OoM | 21,645 | 92,214 | OoM | 20,967 | 89,456 |
| | extrMin | 5 | 157(152) | 14,713 | 63,329 | 26(23) | 2,015 | 8,603 | 24(21) | 1,930 | 8,267 |
| | | 6 | OoM | 23,511 | 101,429 | 44(39) | 3,022 | 12,905 | 40(35) | 2,914 | 12,474 |
| | | 12 | – | – | – | 487(459) | 14,254 | 60,615 | 441(414) | 13,921 | 59,285 |
| | | 13 | – | – | – | 576 | 17,094 | 72,634 | 535 | 16,711 | 71,102 |

of computing bounds by discovering and adding new elements to a partial bound until nothing new can be discovered, quickly converges to termination in these

Table 2: Tight field bounds computation times and achieved speed-ups.

| **LList** | **S10** | **S12** | **S15** | **S17** | **S20** |
|---|---|---|---|---|---|
| TACO(\|\|) | 7.5 | 10.7 | 29.0 | 42.6 | 66.1 |
| TACO(s) | 122.0 | 231.8 | 777.4 | 1204.7 | 1932.5 |
| BU | 1.3 | 2.0 | 3.4 | 5.3 | 11.5 |
| speedup(\|\|) | 5.7X | 5.3X | 8.3X | 8.0X | 5.7X |
| speedup(s) | 91.7X | 114.1X | 224.6X | 227.7X | 166.8X |
| **AList** | **S10** | **S12** | **S15** | **S17** | **S20** |
| TACO(\|\|) | 15.9 | 29.8 | 73.0 | 120.3 | 2174.8 |
| TACO(s) | 381.4 | 807.9 | 2153.8 | 3638.0 | 67936.0 |
| BU | 2.0 | 2.5 | 4.9 | 8.6 | 16.1 |
| speedup(\|\|) | 7.6X | 11.8X | 14.7X | 13.9X | 134.9X |
| speedup(s) | 184.2X | 319.3X | 435.9X | 423.0X | 4217.0X |
| **CList** | **S10** | **S12** | **S15** | **S17** | **S20** |
| TACO(\|\|) | 35.6 | 64.2 | 110.7 | 176.3 | 4634.6 |
| TACO(s) | 981.1 | 1881.9 | 3331.1 | 5386.0 | 145106.0 |
| BU | 2.4 | 4.5 | 12.0 | 54.5 | 2831.2 |
| speedup(\|\|) | 14.6X | 14.0X | 9.2X | 3.2X | 1.6X |
| speedup(s) | 402.0X | 410.8X | 276.8X | 98.7X | 51.2X |
| **AVL** | **S10** | **S12** | **S15** | **S17** | **S20** |
| TACO(\|\|) | 64.6 | 141.9 | 465.9 | 2437.7 | 5939.5 |
| TACO(s) | 1893.7 | 4323.3 | 14645.6 | 77536.6 | 187161.0 |
| BU | 8.1 | 23.0 | 111.4 | 1078.0 | 8562.2 |
| speedup(\|\|) | 7.8X | 6.1X | 4.1X | 2.2X | 0.6X |
| speedup(s) | 231.2X | 187.3X | 131.4X | 71.9X | 21.8X |
| **TSet** | **S10** | **S12** | **S15** | **S17** | **S20** |
| TACO(\|\|) | 76.0 | 145.6 | 258.2 | 872.8 | 2335.4 |
| TACO(s) | 2434.9 | 4411.4 | 8005.7 | 27538.8 | 74134.6 |
| BU | 4.8 | 10.3 | 39.1 | 168.6 | 527.6 |
| speedup(\|\|) | 15.6X | 14.0X | 6.5X | 5.1X | 4.4X |
| speedup(s) | 458.9X | 425.4X | 204.4X | 163.2X | 140.4X |
| **BHeap** | **S10** | **S12** | **S15** | **S17** | **S20** |
| TACO(\|\|) | 115.9 | 188.3 | 345.0 | 1119.7 | 3224.0 |
| TACO(s) | 3505.6 | 5747.1 | 10759.1 | 35409.9 | 102496.0 |
| BU | 4.4 | 9.1 | 23.8 | 80.7 | 243.9 |
| speedup(\|\|) | 26.0X | 20.4X | 14.4X | 13.8X | 13.2X |
| speedup(s) | 786.0X | 625.3X | 452.0X | 438.6X | 420.1X |

cases. Third, some structures with relatively weak invariants also had good running times (AList, in particular), when compared to other case studies. Although the invariants in these cases are weaker, which intuitively would lead to more expensive tight bounds computations, these structures have fewer fields, so the state space to be covered to compute tight bounds is significantly smaller than that of more complex structures.

All the experiments in this section can be reproduced following the instructions available at [1].

*Threats to Validity.* Our experimental evaluation is limited to data structures. From the vast domain of data structures, we have selected a few ones that we consider representative for several reasons: they are often used as case studies in the evaluation of other software analysis tools [6, 9, 18, 28], their invariants have varied complexity (which is a dimension that affects tight bounds' size, and thus their computation), some are acyclic and others are not (which shows that the encoding we make in CBMC is quite general), etc. We consider this is a good menu, representative of a wider class of data structures.

Our approach to capture both the Build* and Gen&Filter strategies might have accidentally favored our technique. We tried different alternatives for capturing Build* and Gen&Filter, in particular with different ways of writing the `repOK` routines (which affected running times). We took the best alternative found for each case, to perform the comparison. In the case of Build*, we took the smallest number of builder routines that guaranteed producing *all* (bounded) structures, since this is a factor that impacts running times. All structures with the exception of CList and TSet required just the `add` routine, while these two also needed a `remove` routine, to guarantee generation of all structures.

Regarding variance across cluster runs, different schedulings indeed yield slightly different timings. Since the granularity of individual analyses is fine, differences are typically small. However, they grow with the scope (e.g., usually smaller than 5% for scope sizes below 10, but up to 15% for the largest sizes). We used the average of 3 runs to reduce the effect of variance in the experiments.

Finally, we did not prove our implementations correct, so our results may be affected by errors in our implementations. We checked consistency of the results across different techniques and tools to confirm that bounds were correctly computed, and verification was bounded complete in all cases.

### 6 Related Work

Automated analysis of code handling dynamic data structures has been the focus of various lines of research, including separation logic based approaches [5], approaches based on combinations of testing and static analysis [22], various forms of model checking including explicit state model checking [27], symbolic execution based model checking [23] and SAT-based verification [14, 13]. The approach that we refer to as Build*, producing nondeterministic structures by using insertion routines, has been used in some of these approaches, including [22, 11]. The "generate & filter" mechanism, on the other hand, is more often employed in modular (assume-guarantee) verification. In particular, the *lazy initialization* approach, whose symmetry breaking we borrowed for "generate & filter" in this paper is used in [19], among others. However, in SAT-based bounded model checking, with tools such as [20], "generate & filter" is not reported as an analysis option for dynamic data structures. The use of tight bounds to improve analysis has been used previously to improve test generation and bounded verification for JML-annotated Java programs [14, 13]. The setting is however different from that of CBMC, due to the relational program (and heap state) semantics, which enabled them to exploit tight bounds directly at the propositional encoding level. Tight bounds have also been used for improving symbolic execution based model checking [15, 26]. Again, the context is different, since these approaches that essentially "walk" the code (either concretely or symbolically), can exploit tight bounds more deeply [26], also obtaining greater profits.

We have also reported a novel technique to *compute* tight bounds. This algorithm is inspired in the work of [24] about black-box test input generation using SAT. Our work is also closely related to [14, 13]. The approach to compute tight field bounds presented in [14, 13] as part of the TACO tool, performs a very large number of independent SAT queries to compute bounds, and thus requires a cluster of workstations to do so effectively (we compared with this approach in the paper). Another alternative approach to compute tight field bounds is presented in [25], but requires structure specifications to be provided in a Separation Logic flavor [21] to compute field bounds.

### 7 Conclusions

We have investigated the use of tight field bounds in the context of SAT-based bounded model checking, more concretely, in (assume-guarantee) verification of C code, using CBMC. We showed that, in this context, and in particular in the verification of programs dealing with linked structures, an approach based on nondeterministically generating structures, and then "filtering out" ill-formed ones, can be more efficient than the more traditional approach of repeatedly using data structure builders, especially when tight bounds are exploited. We have performed a number of experiments that confirm that this alternative approach allows CBMC to consider larger input sizes as well as to detect bugs that could not be detected without using bounds.

Since the approach depends on precomputing tight field bounds, we have also studied this problem, providing a novel algorithm for tight field bound computation. Tight field bounds have proved useful for a number of different analyses, but computing them is costly, and previous field bound computation approaches that performed reasonably did so at the expense of relying on a cluster of workstations to perform the task, or were only applicable to a limited set of class invariants, expressible in separation logic. Thus, while tight field bounds proved to have a deep impact in the previously mentioned automated software analysis techniques, their use has been severely undermined by the necessity of a cluster of computers for their effective computation, or the availability of specifications in separation logic. The algorithm presented in this article allows one to compute tight field bounds on a single workstation more efficiently than the distributed approach on a cluster of 8 quad-core, and therefore makes tight field bounds computation both practical and worthwhile, as part of the above mentioned analyses.
